# In this issue

**Published:** 2022-08

**Prevalence of obesity in treated and untreated patients with attention deficit hyperactivity disorder. *A meta-analysis***

AlAhmari & Uddin discuss and summarize the difference in obesity rate in treated and untreated attention deficit hyperactivity disorder (ADHD) patients to evaluate the influence of ADHD medication on weight status in these individuals. Using PubMed, Cochrane Library, and Google Scholar databases were searched for eligible articles from January to December 2020 using the following medical subject headings. A total of 19,449 study participants included in selected 8 studies were assessed with respect to the prevalence of obesity in medicated and unmedicated subgroups of ADHD patients. This result suggests that the treatment is not only important for controlling ADHD manifestations but is also associated with lower body mass index.

***see page 873***

**Figure:**
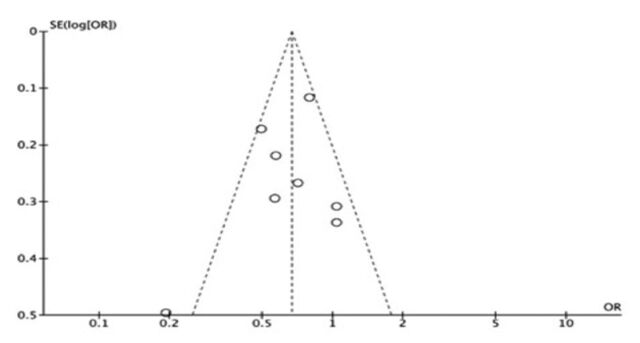
Funnel plot-publication bias

