# Supplementary material for: In this issue
Source: Saudi Med J. 2022 Aug;43(8):871. (PMC9749669)
Supplement: Related texts

# Abstract: Original Articles

**Effects of different fresh gas flows on carboxyhemoglobin levels: non-invasive carbon monoxide monitoring. *A randomized clinical trial***

Köksal et al observe a total of 64 patients the effect of different fresh gas flows (FGF) on carboxyhemoglobin (COHb) levels non-invasively and continuously and to determine the contribution of the smoking status to intraoperative carbon monoxide (CO) accumulation and respiratory complications. The mean COHb values were significantly higher in Group L between 30th and 210th minutes. Furthermore, in Group L, intraoperative COHb levels were significantly higher in smokers compared to non-smokers in all periods. In group H, no difference was observed between smokers and non-smokers in terms of COHb levels after 60 minutes and also preoperative COHb levels of the patients developed respiratory complication was higher. If the CO2 absorbent is properly preserved in patients who are administered low-flow anesthesia, there will be no risk of CO accumulation even in chronic smokers.

***see page 891***

**Detection of erythroid progenitors and erythrocytopathies in patients with severe COVID-19 disease**

Allahyani et al assess the effect of severe acute respiratory syndrome coronavirus-2 infection on erythropoiesis and red blood cells surface markers by evaluating erythroid progenitor cells (CD [cluster of differentiation]71_+_/CD235a_+_) and RBC surface markers (CD235a and CD36), together with various hematological parameters of 47 participants. The data showed that more than half of the COVID-19 patients were anemic (64%). The expansion of erythroid progenitors and alteration of RBC surface markers can contribute to erythrocytopathies observed in severe COVID-19 patients and can therefore be used as prognostic factors.

***see page 899***

**Figure:** Dot plots represent the percentage of CD71 and CD235a in the samples obtained from the healthy individuals and COVID-19 patients.

**Awareness of medical law among health care practitioners in Saudi Arabia**

Bin Shihah et al determine the level of awareness of medical law in 2020-2021 via a survey including 750 healthcare practitioners and to identify factors that influence that level of awareness in Saudi Arabia. Majority of enrolled healthcare practitioners had poor awareness of medical law (approximately 97%). Only 1.5% had adequate awareness of medical law, and only 1.5% had moderate awareness. Awareness of medical law among healthcare practitioners is limited in Saudi Arabia. Professional medico-legal education should be part of required competencies for undergraduate and postgraduate levels of medical education. Health care practitioners must be educated with laws and regulations of practicing health professions in the country.

***see page 899***
